# Multiple sclerosis and genetic polymorphisms in fibrinogen-mediated hemostatic pathways: a case–control study

**DOI:** 10.1007/s10072-021-05608-1

**Published:** 2021-09-24

**Authors:** Gianmarco Abbadessa, Giuseppina Miele, Andrea Di Pietro, Maddalena Sparaco, Raffaele Palladino, Ignazio Armetta, Giovanna D’Elia, Francesca Trojsi, Elisabetta Signoriello, Giacomo Lus, Luigi Lavorgna, Simona Bonavita

**Affiliations:** 1 Department of Advanced Medical and Surgical Sciences, University of Campania “Luigi Vanvitelli”, Naples, Italy; 2 Department of Public Health, University Federico II, Naples, Italy; 3 Department of Primary Care and Public Health, Imperial College London, London, UK; 4 Clinical and Molecular Pathology, Department of Precision Medicine, University of Campania Luigi Vanvitelli, Naples, Italy

**Keywords:** Multiple sclerosis, Fibrinogen, Coagulation, Polymorphisms

## Abstract

**Introduction:**

Blood coagulation constituents might exert immunomodulatory functions in the CNS and may trigger neuroinflammation and demyelination. We evaluated whether particular single-nucleotide polymorphisms (SNPs), thought to be involved in fibrinogen-mediated hemostatic pathways, are overrepresented in patients with MS compared with controls.

**Methods:**

The case–control study consisted of 119 MS patients recruited consecutively at our clinic, and 68 healthy controls. Afterwards, we created a cumulative genetic risk score (CGRS) which included the 5 selected hemostatic risk alleles (Beta-Fibrinogen 455G/A, Glycoprotein IIIa P1A2, Factor V Leiden, Factor V H2R, and Prothrombin 20210G/A). Multivariate ordinal logistic regression and multivariate multinomial logistic regression were applied to evaluate the effect of CGRS on MS susceptibility.

**Results:**

The FGB 455 G/A and Factor V H1299R variants might be associated with MS status, in the recessive and dominant model, respectively. A cumulative association of the five SNPs investigated with the disease was observed.

**Discussion:**

We found that MS patients carried more pro-hemostatic variants than healthy controls. An increasing number of unfavorable alleles might increase the likelihood of being in the MS group, in the cumulative analysis. Our findings encourage to evaluating these variants in a larger population-based cohort.

**Supplementary Information:**

The online version contains supplementary material available at 10.1007/s10072-021-05608-1.

## Introduction

Multiple sclerosis (MS) is a chronic inflammatory autoimmune disease that affects the central nervous system (CNS) causing demyelination and axonal damage [1]. Several genetic and environmental factors have been linked to the disease and adaptive immunity, and autoimmune phenomena seem to play a pivotal role in its pathogenesis [1]. Since its first pathological description, vascular alterations have been described. Above all, blood–brain barrier (BBB) leakage, fibrin deposition in the perivascular space, vessel occlusion, and cerebral hypoperfusion have been better characterized by subsequent studies [2]. Furthermore, increasing evidence has suggested that blood coagulation constituents might exert immunomodulatory functions in the CNS with critical consequences in fostering and amplifying neuroinflammation and the demyelinating process [3, 4]. The strongest evidence is available for fibrin(-ogen). It leaks out through the damaged BBB at the earliest stage of MS lesion, preceding demyelination and axonal loss, and spatially correlates with areas of demyelination and axonal damage [5]. In experimental allergic encephalitis (EAE), fibrinogen specifically promotes the activation of macrophages/microglia and favors the recruitment of encephalitogenic T cells in the CNS [6, 7]. Moreover, fibrin-targeting immunotherapy can be selective and efficacious in suppressing neuroinflammation and neurodegeneration in EAE [8].

Due to the key role displayed by fibrin(-ogen) in the extensive cross talk between hemostasis and neuroinflammation, our purpose was to determine whether particular single-nucleotide polymorphisms (SNPs), thought to be involved in fibrinogen-mediated hemostatic pathways, are overrepresented in patients with MS compared with controls. Furthermore, in order to identify genetic factors predicting a worse outcome in MS patients, we investigated whether the frequency of the investigated SNPs was associated with clinical and radiological outcomes at diagnosis.

## Methods

### Patients

This unmatched case–control study consists of 119 relapsing–remitting (RR)-MS patients recruited consecutively at our clinic, and 68 healthy controls. Data on patient characteristics at diagnosis, including Expanded Disability Status Scale (EDSS) and radiological features (number of T2 lesion ≧ 9, presence of spinal cord involvement, and presence of contrast-enhancing lesions), were collected. Exclusion criteria for patients were as follows: history of cerebral and/or cardiovascular ischemic events, thrombotic or bleeding disorders, chronic autoimmune diseases. Controls were recruited among hospital employees. The control group consisted of volunteers without a self-reported history of cerebrovascular or cardiovascular disease, thrombotic or bleeding disorders, or autoimmune chronic disease. As genetic polymorphisms vary between races and populations, all the controls had the same ethnic (Caucasian) and geographic background (people originating from Campania, Southern Italy) as patients.

### Selection of SNPs and genotyping

The assay to assess the genetically determined cardiovascular risk was the *Cardiovascular Disease (CVD) 14* (Nuclear Laser Medicine, Naples, IT; for more details, see Supplementary Material). It allows for the identification of the most relevant gene mutations and polymorphisms involved in arterial and deep venous thrombosis (Factor V R506Q and H1299R, Prothrombin G20210A, Methylenetetrahydrofolate Reductase (MTHFR) C677T and A1298C, Cystathionine Beta Synthase (CBS) 844ins68, Plasminogen Activated Inhibitor (PAI-1) 4G/5G, Angiotensin-Converting Enzyme (ACE) Ins/Del, Angiotensin (AGT) g.9543 T > C (M235T), Glycoprotein IIIa (GPIIIa) T1565C HPA-1 a/b, Angiotensin Receptor 1 (ATR-1) A1166C, β-fibrinogen (FGB) G455A, and Factor XIII g.7130G > T (V34L) and in the cholesterol metabolism (Apolipoprotein E (ApoE)). Among the 14th SNPs included in the CVD 14 assay, five SNPs were selected based on the underlying biological plausibility discussed above: FGB 455 G/A (rs#1,800,790), Factor V 1691 G/A (rs#6025), Factor V 1299 H/R (#rs770011773), Prothrombin 20,210 G/A (rs#1,799,963), GpIIIa 1565 T/C (#rs59189) (Table 1). DNA extraction from non-coagulated blood was performed for all samples using the DNA extraction kit, according to the manufacturer’s instructions (see Supplementary Material for details). DNA target was amplified with multiplex PCR using biotinylated primers in a thermal cycler under the following conditions: 95 °C for 2 min, 95 °C for 30 s, followed by denaturation at 60 °C for 30 s, annealing at 72 °C for 45 s, and extension at 72 °C for 5 min for a total of 35 cycles. The amplification products were analyzed by means of 2% agarose gel containing ethidium bromide (Sigma-Aldrich) as intercalating DNA agent. Ethidium bromide is the most used nucleic acid stain for agarose gel electrophoresis. The DNA bands were detected on a UV light box. Detection is based on the reverse-hybridization principle and was performed by using automatic instrument Profi-Blot T30/T48. Biotinylated PCR products were hybridized with allele-specific oligonucleotide probes immobilized as an array of parallel lines on membrane-based strips. The exact match between probes and amplified product generates a signal exploiting the bond between biotin and streptavidin conjugated with alkaline phosphatase and a subsequent color developer that allows for colorimetric detection.

**Table 1:** Description of the selected single-nucleotide polymorphisms (SNPs)

Hemostatic risk allele	Approved name	Approved symbol	HGNC ID	Chromosomal location	RS	Point mutation nucleotide	Allele type
Beta-Fibrinogen 455 G/A	Fibrinogen beta chain	FGB	3662	4q31.3	rs1800790	455	G/A
GpIIb/IIIa PIA2	Integrin subunit beta 3	ITGB3	6156	17q21.32	rs5918	1565	T/C
Factor V Leiden	Coagulation factor V	F5	3542	1q24.2	rs6025	506	G/A
Factor VH2R	Coagulation factor V	F5	3542	1q24.2	rs1800595	1299	T/C
Prothrombin G20210A	Coagulation factor II, thrombin	F2	3535	11p11.2	rs1799963	20,210	G/A

*A*, adenine; *C*, cytosine; *G*, guanine; *GpIIIa*, glycoprotein IIIa; *HGNC*, HUGO Gene Nomenclature Committee; *RS*, reference sequence; *T*, thymine

### Cumulative genetic risk score

Risk associations between each risk genotype and MS susceptibility were tested under three different genetic models: dominant, additive, and recessive models [9] (as shown in Table 3). Afterwards, for each SNP, the genotypes were coded as 0, 1, or 2 indicating the number of hemostatic risk alleles in the genotype in order to create a cumulative genetic risk score (CGRS) which included the 5 selected hemostatic risk alleles (rs#1,800,790, rs#6025, #rs770011773, rs#1,799,963, #rs59189) (Table 1). CGRSs were calculated using the unweighted method, mirroring previous studies [10]. The unweighted CGRS of an individual is the sum of disease alleles from five SNPs obtained by adding coded genotypes (possible score range of 0–10). To avoid any bias due to missing data, samples with one or more missing genotypes were not included in the genetic risk score calculations. For the unweighted genetic score analysis, genotypes from 98 cases and 60 healthy controls were considered.

### Statistical analysis

For each SNP, Hardy–Weinberg equilibrium was tested in the control sample by applying chi-square test. All the demographic characteristics between MS cases and healthy controls were evaluated by applying a *t*-test for continuous data and chi-square test for discrete data. A multivariable logistic regression model was employed to assess the association between MS status and each polymorphism in the recessive and dominant model, while a multivariable multinomial logistic regression model was employed to assess the association between MS status and each polymorphism in the additive model. It is difficult to reach stringent measures of statistical significance with a small sample size, and the fairly conservative multiple testing approach, Bonferroni correction, may increase type II error (false negatives); therefore, to overcome these shortcomings, we used the unweighted CGRS. Distributions of CGRS between MS cases and controls were compared by applying nonparametric Mann–Whitney *U* test. Moreover, *t*-test was applied to compare average CGRS between cases and controls. We employed an ordinal logistic regression model to assess the association between CGRS and MS status. Then, CGRS was divided into three groups based on the number of risk alleles in subjects (no risk: no risk alleles; low risk: one or two risk alleles; high risk: three or four risk alleles). The group with no risk alleles was used as the base outcome in the multinomial logistic regression. Testing for dose–response or testing the risk factor trend on the outcome was also applied. All the regression models were adjusted according to age and gender. Lastly, to investigate in MS patients whether the frequency of those SNPs is associated with clinical and radiological outcomes at diagnosis, a multivariable logistic model was applied. Bonferroni adjustments were performed when appropriate. Bonferroni adjustments were performed for the Mann–Whitney *U* test applied to describe distributions of CGRS between MS cases and controls and for the *t*-test that was applied to compare average CGRS between cases and controls. Concerning the multivariate analyses, the correction for multiple comparisons was not performed for the following reasons: (i) reducing the type I error for null associations, which is the reason to correct for multiple comparisons, increases the type II error for those associations that are not null [11]; (ii) given the exploratory nature of our analyses.

Statistical analyses were performed using Stata (StataCorp. 2019. *Stata Statistical Software: Release 16*. College Station, TX: StataCorp LLC).

## Results

### Association analysis of the five SNPs with MS

Demographic and clinical characteristics of the study sample are reported in Table 2. Controls were significantly younger than cases (*p* value = 0.047). There was no statistically significant difference between the two groups as concerns gender. All SNP variants satisfied Hardy–Weinberg equilibrium in the control sample, with a *p-*value > 0.05. When applying the Hardy–Weinberg equilibrium equation to FBG to the 38 GG and to the 29 GA carriers of the control group, an A allele prevalence of 0.22 can be estimated. This translates into an expected number of 3 AA carriers (1 observed). The frequencies of each variant did not show any significant difference between males and females (Table S1). In Table 3, the allelic and genotypic frequency in MS patients and healthy controls is reported. The *p* values of the chi-square tests displayed in Table 3 for additive model refer to by 2 by 3 contingency tables and do not show the difference in risk carried by the genotype G/A compared to the genotype G/G. Therefore, our results do not suggest that the Beta-Fibrinogen 455 G/A variant is associated according to different transmission models (both additive and recessive). To highlight this, we have performed a 2 by 2 contingency table to evaluate the frequency of G/A compared to G/G between cases and controls. Results of the analysis did not show a difference between these two genotypes, as shown in Table S2a (in Supplemental Material).

**Table 2:** Demographic and clinical data of MS patients and healthy controls

	MS^§^ patients (*n*) 119	Healthy controls (*n*) 68	*P* value
Age (years) Mean (SD)	45.394 (12.06)	41.808 (17.02)	0.047
Male sex *N* (percentage)	43.7%	49.7%	0.705
Age of onset (years) Mean (SD)	32.294 (11.17)	**-**	**-**
EDSS^†^ at diagnosis Mean (SD)	2.424 (1.24)	**-**	**-**
More than 9 lesions in T2 at diagnosis *N* (percentage)	74 (64.5%)	**-**	**-**
Spinal cord involvement at diagnosis *N* (percentage)	81 (70%)	**-**	**-**
Gd + ^‡^ lesion/s at diagnosis *N* (percentage)	26 (25%)	**-**	**-**

*MS*, multiple sclerosis; *EDSS*, Expanded Disability Status Scale; *GD* + , gadolinium +

**Table 3:** Allelic and genotypic frequency in MS patients and healthy controls

SNP^‡^	Additive model	Case/control		Allelic model	Case/control		Recessive model	Case/control		Dominant model	Case/control	
		***N* freq**	***P* value (**chi-square)		**Freq**	***P* value (**chi-square)		***N* freq**	***P* value (**chi-square)		***N* freq**	***P* value (**chi-square)
Beta-Fibrinogen rs#1,800,790	**GG** **GA** **AA**	63 (55.75%)/38 (55.88%) 36 (31.85%)/29 (42.64%) 14 (12.38%)/1 (1.47%)	**0.024**	**G** **A**	71.68%/77.21% 28.3%/22.79%	0.725	**GG/GA** **AA**	99 (87.61%)/67 (98.53%) 14 (12.39%)/1 (1.47%)	**0.010**	**GG** **GA/AA**	63 (55.75%)/ 38 (55.88%) 50 (44.25%)/ 30 (44.12%)	0.986
GpIIIa #rs5918	**TT** **TC** **CC**	70 (67.96%)/48 (77.41%) 25 (24.27%)/12 (19.35%) 8 (7.76%)/2 (3.22%)	0.298	**T** **C**	80.1%/87.1% 19.9%/12.9%	0.129	**TT/TC** **CC**	93 (92.08%)/60 (96.77%) 8 (7.92%)/2 (3.23%)	0.225	**TT** **TC/CC**	68 (67.33%)/ 48 (77.42%) 33 (32.67%)/14 (22.58%)	0.167
Factor V Leiden rs#6025	**GG** **GA** **AA**	N/A	N/A	**G** **A**	97.9%/97.79% 2.1%/2.21%	0.955	**GG/GA** **AA**	N/A	N/A	**GG** **GA/AA**	114 (95.8%)/65 (95.59%) 5 (4.20%)/3 (4.41%)	0.946
Factor V H2R #rs1800595	**HH** **HR** **RR**	N/A	**N/A**	**HR**	91.53%/96.97% 8.47%/3.03%	0.540	**HH/HR** **RR**	N/A	N/A	**HH** **HR/RR**	99 (83.90%)/62 (93.94%) 19 (16.10)/4 (6.06)	**0.048**
Prothrombin 20,210 G/A rs#1,799,963	**GG** **GA** **AA**	N/A	N/A	**G** **A**	95.8%/98.53% 4.2%/1.47%	0.145	**GG/GA** **AA**	N/A	N/A	**GG** **GA/AA**	109 (91.60%)/66 (97.06%) 10 (8.40%)/2 (2.94%)	0.143

*SNP*, single-nucleotide polymorphism; *A*, adenine; *C*, cytosine; *G*, guanine; *GpIIIa*, glycoprotein IIIa; *H*, histidine; *R*, arginine; *rs*, reference sequence; *T*, thymine

Concerning the FV H1299R, FV Leiden, and Prothrombin G20210A variants, no subjects in our population carried the homozygotic genotype for the risk alleles; therefore, the additive model, as well as the recessive one, is not applicable.

Results from the association analysis between individual SNPs and MS susceptibility revealed those carrying the genotype AA of the FGB polymorphism were likely to have MS as well, in the additive model (OR 8.994; 95% CI 1.108–72.976; *p* = 0.040) and in the recessive model (OR 10.101; 95% CI 1.265–80.646; *p* = 0.029), after adjustment for age and gender; however, those carrying the genotype GA did not show an increased likelihood of being in the MS status in the additive model (Table S2b). Therefore, the Beta-Fibrinogen 455 G/A polymorphism might be associated to the MS status only according to a recessive pattern of inheritance. Although more frequent in MS patients, none of the other 4 SNPs was significantly associated with MS status, after adjustment for age and sex, except for the Factor V H1299R that was significantly associated with MS status in the dominant model (OR 3.130; 95% CI 1.012663–9.679648; *p* value = 0.048).

### Cumulative allele score analysis

**Fig. 1:**
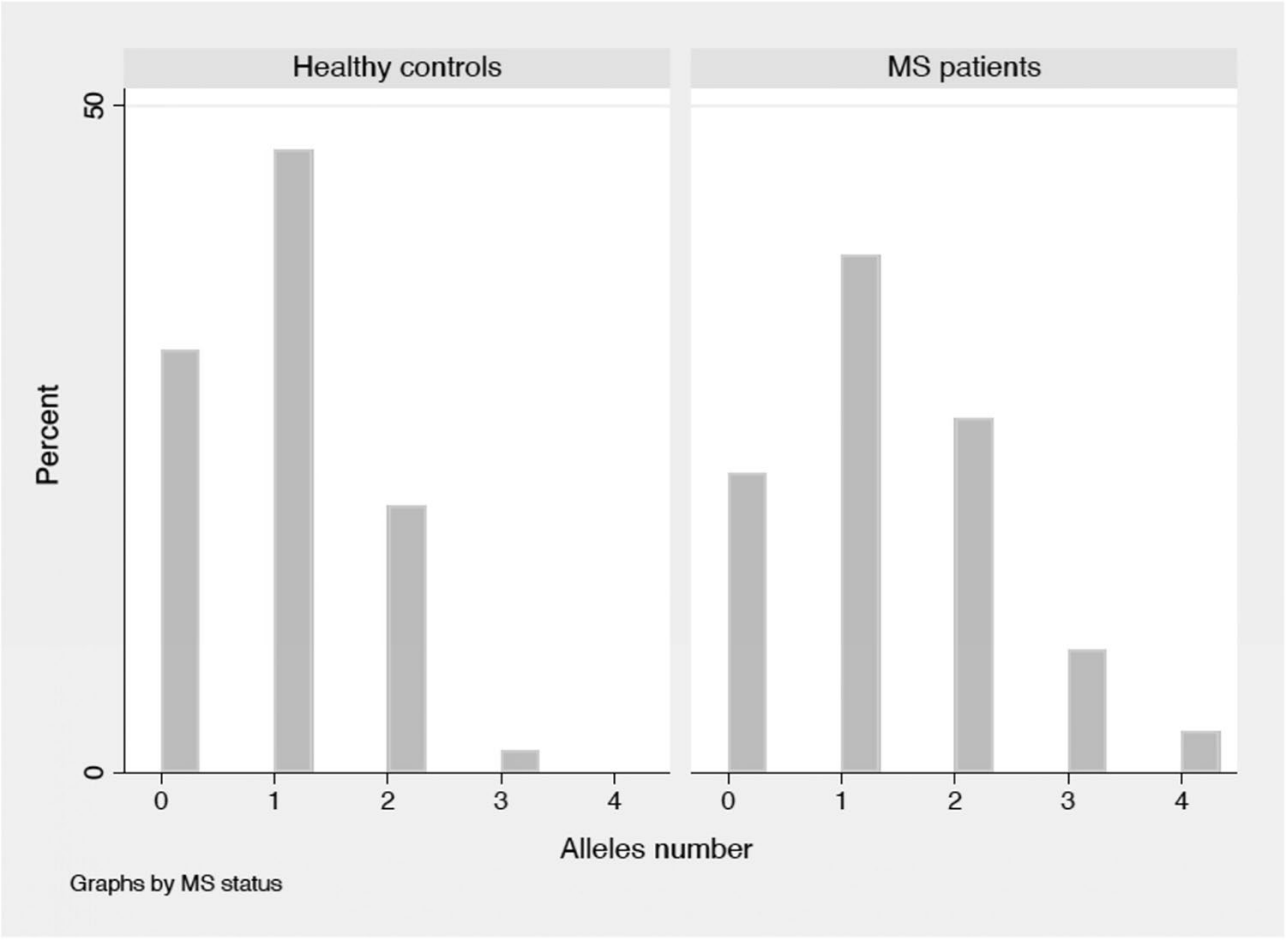
Distribution of CGRS between MS patients and healthy subjects. The distribution of the cumulative genetic risk score in multiple sclerosis patients and healthy controls is represented. Allele number: number of risk alleles carried by subjects (0, 1, 2, 3, or 4)

We examined the combined effect of these five thrombotic-related SNPs on MS susceptibility by creating a CGRS. In cases, the distribution of CGRS showed a significant shift towards higher values compared to controls (Mann–Whitney *U* test *p*-value < 0.05). However, its significance did not withstand adjustment for multiple testing using the Bonferroni correction. The average (± SD) of CGRS among MS cases (1.273 ± 1.025 SD) was significantly higher than controls (0.822 ± 0.820 SD; *t*-test *p*-value = 0.0029), comparing the two groups, and its significance withstood adjustment for Bonferroni correction (*p* = 0.0145). The distribution of CGRS between cases and controls is graphically showed in Fig. 1. As summarized in Table 4, multivariate ordinal logistic regression found a significant association between CGRS and MS status (*p* = 0.010). Then, individuals were grouped into three categories according to the weighted risk scores, no risk (CGRS = 0), low risk (CGRS = 1–2), and high risk (CGRS = 3–4). The effect sizes of the low and high groups were estimated by taking the no risk group as reference (Table 4). We observed a difference in the likelihood of being in the MS status for subjects in both categories of risk (low risk: RRR 2.193; 95% CI 1.075–4.472; *p* = 0.031; high risk RRR 8.360; 95% CI, 1.497–107.503; *p* = 0.047). To illustrate the effect per increase 1 risk allele, we calculated the odds ratio (OR) for trends. As the CGRS increased, the OR increased (*p* = 0.002). The ORs increased to the values of 2.126 (95% CI 1.035–4.283; *p* value = 0.0354) and 12.913 (95% CI 1.301–128.122; *p* = 0.0046) for subjects carrying respectively 1–2 and 3–4 risk alleles (Table 4).

**Table 4:** Cumulative genetic risk score (CGRS) in MS patients compared to controls

Ordered logistic regression						
	*Adjusted OR*		*p value*		*[95% conf. interval]*	
Multiple sclerosis	2.217		**0.010**		1.207–4.069	
1 risk allele	− 0.203		-		− 1.187–0.780	
2 risk alleles	1.431		-		0.424–2.439	
3 risk alleles	3.169		-		2.030–4.307	
4 risk alleles	4.632		-		3.123–6.141	
Multinomial logistic regression						
Risk alleles	*Adjusted RRR*			*p value*	*[95% conf. interval]*	
0	Base outcome					
1–2	2.193			**0.031**	1.075–4.472	
3–4	8.360			**0.047**	1.497–107.503	
Test for trend						
Risk alleles	*OR*	*p value*				*[95% conf. interval]*
0–1	1	-				-
2	2.106	**0.0354**				1.035–4.283
3–4	12.913	**0.0046**				1.301–128.122
*P* trend: 0.002						

*OR*, odds ratio; *RRR*, relative risk ratio

We did not find any significant association between the separate or combined effects of the genotypes and clinical or radiological outcomes of MS patients at diagnosis.

## Discussion

In this case–control study involving subjects originating from Campania, we investigated the association between five common genetic polymorphisms involved in fibrinogen-mediated hemostatic pathways with MS status. Our results revealed that FGB 455 G/A and Factor V H1299R variants might be associated with MS status, in the recessive and dominant model, respectively.

Our population of healthy subjects had a similar allele frequency of the 5 tested SNPs to that reported in healthy subjects from Europe, Italy (Tuscany), and Campania [12, 13], see Table S3 (Supplemental Material).

It is becoming increasingly clear that the genetic predisposition to common diseases is multifactorial, often resulting from multiple low-penetrance variants. The possible biological effect of the five markers under study in enhancing and sustaining neuroinflammation is supported by a growing body of evidence revealing the role of the fibrinogen-mediated hemostatic pathways in the pathogenesis of MS. In detail, we have studied the polymorphisms involved in the regulation of (a) fibrinogen interaction with its platelet receptor (GP IIIa P1A2); (b) fibrinogen synthesis (Beta-Fibrinogen 455 G/A); and (c) fibrinogen cleavage into fibrin (Factor V Leiden, Factor V H2R, Prothrombin 20,210 G/A).

The GP IIb/IIIa integrin is the most abundant platelet-specific glycoprotein, functioning as a receptor for ligands such as fibrinogen and von Willebrand factor, promoting platelet activation and aggregation [14]. The GPIIIa P1A variant may potentially influence both activation of the GP complexes and platelet aggregation [15, 16]. The interplay between activated platelets, endothelial cells, and infiltrating leukocyte regulates local inflammatory response and is an underlying mechanism of BBB dysfunction [17]. Several experiments have revealed an increased platelet activation, adhesiveness, and aggregation in RRMS patients [17–20]. Moreover, enhanced activation of GPIIb/IIIa, augmented formation of platelet aggregates, and increased platelet adhesiveness [21, 22] were found in secondary progressive MS patients.

The other four SNPs (Factor V 1691 G/A, Factor V 1299 H/R, Prothrombin 20,210 G/A, FGB 455 G/A) influence the transcription of genes encoding for proteins involved in secondary hemostasis. Factor V Leiden and Factor V H1299R mutations may cause activated protein C (aPC; Factor V and Factor VIII inhibitor) resistance by reducing the susceptibility of activated Factor V and activated Factor VIII to aPC-mediated inactivation. The Factor II G20210A polymorphism in the 3′ untranslated region of the prothrombin gene is associated with an increased level of thrombin activity [24], whereas the FGB 455 G/A polymorphism is associated with a significantly higher fibrinogen plasma level [25]. The effect on coagulation cascade of aPC-resistance, of augmented thrombin activity, and increased fibrinogen levels is the induction of a state of hypercoagulability resulting in an increased fibrin deposition [23, 24].

Significantly higher plasma levels of prothrombin have been found in MS patients compared to healthy controls [25]. Moreover, RRMS patients showed an accelerated thrombin generation compared to both PPMS and healthy controls, probably depending on the active proinflammatory state [26]. In a recent study, plasma levels of PC pathway proteins have been associated with neuroradiological measures of atrophy (volumes of total gray matter, thalamus, cortex, deep gray matter, and whole brain) [27]. Further evidence corroborating a role for thrombin and PC pathway in MS pathogenesis derives from neuropathological and experimental studies. Proteomic analysis of chronic active lesions revealed the concomitant presence of tissue factor (the initiator of coagulation cascade) and protein C inhibitor (PCI), suggesting that suppressed protein C pathway and consequently enhanced thrombin formation may play a role in the chronic neuroinflammatory process [28].

Fibrin(-ogen) was shown to be deposited in MS lesions in both RR and progressive forms. It colocalizes with microglia/macrophages and perivenous demyelination in all active lesions in RRMS, whereas it is diffusely distributed in chronically active and inactive lesions overlapping with astrocyte and axonal processes in progressive forms [29, 30].

Recently, Lee and colleagues investigated the spatial–temporal dynamics of fibrinogen deposition in marmoset EAE by creating a radiology-to-pathology linkage. They revealed fibrin deposition in the non-demyelinated inflammatory nodules, an early MS pathological finding without a magnetic resonance imaging (MRI) counterpart. Moreover, they detected fibrin(-ogen) at the border of chronic active but not in chronic inactive plaques, suggesting that fibrinogen may play a central role in sustaining chronic inflammation [5]. Moreover, fibrin(-ogen) deposition, colocalizing with axons in chronically active and inactive lesions and with neuronal loss in cortical gray matter, has revealed a linkage even with some pathological neurodegeneration findings in MS [31].

All these findings suggest the involvement of coagulation molecules, particularly fibrinogen, in the pathogenesis of MS. The complex interaction between the hemostatic and neuroinflammatory effectors seems to have a critical role in the demyelinating process since its earliest stage. An unbalanced genetic profile towards a pro-thrombotic state might constitute a predisposing condition for MS development. Indeed, the cumulative effect of the analyzed pro-thrombotic variants might influence BBB stability and, therefore, might promote coagulation molecule extravasation, deposition, and activation, which may foster the neuroinflammatory process. Due to the minimal contribution of each variant to MS susceptibility, two (FGB G455A and Factor V H1299R) of the five genetic polymorphisms under study were found to be weakly associated with MS status. In light of the synergic effects of the five SNPs in enhancing a pro-hemostatic condition, we evaluated the cumulative effects of the five genetic polymorphisms by computing a CGRS. As this is the first study to explore the association between these SNPs and MS susceptibility, CGRS was calculated using the unweighted method. Indeed, to use the weighted method, we need the true OR [10]; these estimates are not currently known. We found that MS patients carried more pro-hemostatic variants than healthy controls and that an increasing number of unfavorable alleles might increase the likelihood of being in the MS group, in the cumulative analysis; however, we should highlight that the controls were younger than cases, potentially due to an enrollment bias and this could have influenced our results. Despite this, the significance withstood the adjustment for age and sex.

We did not find correlation between the risk genotypes and the clinical-radiological outcomes of MS patients at diagnosis.

Finally, we would also highlight that none of the 5 tested SNPs (FGB 455 G/A (rs#1,800,790) on chromosome 4; GpIIIa 1565 T/C (#rs59189) on chromosome 17; Factor V 1691 G/A (rs#6025) and Factor V 1299 H/R(#rs770011773) on chromosome 1; Prothrombin 20,210 G/A (rs#1,799,963) on chromosome 11) is in linkage disequilibrium with some of the 200 loci known to be associated with MS [32]. Details are reported in Table S4, S5, S6, S7 and S8, in Supplemental Material.

Therefore, although the predictive values of the considered genetic variants are not backed by a highly significant statistic, our findings do not discount the involvement of these factors in MS pathogenesis and suggest evaluating these variants in a larger population-based cohort. Indeed, identifying genetic variants associated with MS could increase our knowledge of disease mechanisms; this may represent a further step towards personalized medicine by distinguishing patients according to different pathways involved in neuroinflammation.

### Future perspective

This study had an explorative nature and aimed only to describe the frequency distribution of pro-thrombotic polymorphisms among patients with multiple sclerosis and healthy controls. However, since these findings would benefit from an in silico analysis, future studies are needed to assess the impact of the selected variants on the expression profile of related genes and of other genes in the same locus, using RNA expression data from brain, available in public databases.

## Supplementary Information

Below is the link to the electronic supplementary material.

Data on sensitivity and specificity of the Cardiovascular Disease 14 assay and allele frequency in females and males.(DOCX 46 KB)

## Data Availability

The data that support the findings of this study are available from the corresponding author upon reasonable request.

## Declarations

Ethical approval

The ethics committee of the University of Campania Luigi Vanvitelli approved this study (n. protocol 11222/20). The study conforms with the World Medical Association Declaration of Helsinki.
