# Supplementary material for: Multiple sclerosis and genetic polymorphisms in fibrinogen-mediated hemostatic pathways: a case–control study
Source: Neurol Sci. 2021 Sep 24;43(4):2601–9. doi: 10.1007/s10072-021-05608-1 (PMC8918146; doi:10.1007/s10072-021-05608-1)
Supplement: Supplementary file 1 — Data on sensitivity and specificity of the Cardiovascular Disease 14 assay and allele frequency in females and males.(DOCX 46 KB) [file 10072_2021_5608_MOESM1_ESM.docx]

**Supplementary Material**

***Data on sensitivity and specificity of the Cardiovascular Disease 14 assay***

Diagnostic Sensitivity

The diagnostic sensitivity of the kit CVD14 was assessed by analyzing 43 DNA samples positive for the mutations and polymorphisms investigated by the kit.

All the samples were correctly genotyped, resulting in a diagnostic sensitivity of 100%.

Analytical Sensitivity

The analytical sensitivity of the kit CVD 14, expressed as the minimum quantity of the target that can be detected, is equal to 5 ng/μl DNA.

Diagnostic Specificity

The diagnostic specificity of the kit CVD 14 was determined by analyzing 43 samples. All samples were correctly amplified and genotyped. In agreement with these data, the diagnostic specificity is 100%.

**DNA extraction kit (Nuclear Laser Medicine).**

As indicated by the kit instruction: “pipet 25 μl Proteinase K and 200 μl sample into 1,5 ml centrifuge tubes previously marked and incubate 5 min at room temperature. Add 200 μl Lysis Buffer to the samples and vortex, incubate 5 min at room temperature. Incubate samples at 70°C for 10-15 min mixing by vortex two times during incubation. Subsequently, apply the lysate to each spin column in a 2 ml collecting tube, previously marked, and centrifuge 1 min at 11000 x g. Place the spin column in a clean 2 ml collecting tube, add 500 μl Wash Buffer and centrifuge 1 min at 11000 x g. Place the spin column in a clean 2 ml collecting tube, add 600 μl Wash Buffer and centrifuge 1 min at 11000 x g. Place the spin column in a clean 1,5 ml microcentrifuge tube and add 100 μl Elution Buffer, prewarmed at +70°C and incubate at room temperature for 1 min. Centrifuge 1 min at 11000 x g, eluate should appear clear.

***Table S1 - Allele frequency in females e males***

| SNP | Additive model | Famale / Male | | Allelic model | Famale / Male | |
| --- | --- | --- | --- | --- | --- | --- |
|  |  | **N freq** | **P value**  **(**chi-square) |  | **Freq** | **P value**  **(**chi-square) |
| Beta-Fibrinogen G455A  rs#1800790 | **GG**  **GA**  **AA** | 57 (55,33%) / 44 (57,89%)  37 (35,92%) / 27 (35,52%)  9 (8,73%) / 5 (6,57%) | 0,756 | **G**  **A** | 73,3% / 75,66%  26,7% / 24,34% | 0,702 |
| GpIIIa PIA2  #rs59189 | **TT**  **TC**  **CC** | 64 (69,56%) / 45 (70,31%)  24 (26,08%) / 13(20,31%)  4 (4,34%) /  6 (9,37%) | 0,312 | **T**  **C** | 82,61% / 80,47%  17,39% / 19,53% | 0,739 |
| Factor V Leiden  rs#6025 | **GG**  **GA**  **AA** | 101 (98,05%) / 71 (93,42%)  2 (1,94%) / 5 (6,57%)  0 (0%) / 0 (0%) | 0,241 | **G**  **A** | 99,03 % / 96,71%  0,97% / 3,29% | 0,238 |
| Factor V H2R  #rs770011773 | **HH**  **HR**  **RR** | 88 (85,43%) / 70 (92,10%)  15 (14,56%) / 6 (7,89%)  0 (0%) / 0 (0%) | 0,326 | **H**  **R** | 92,72 % / 96,05 %  7,28% /  3,95 % | 0,271 |
| Prothrombin 20210 G/A  rs#1799963 | **GG**  **GA**  **AA** | 96 (93,20%) / 70 (93,33%)  7 (6,79%) / 5 (6,66%)  0 (0%) /0(0%) | 0,954 | **G**  **A** | 96,6 % / 96,67%  3,4% /3.33% | 0,791 |

SNP: Single Nucleotide Polymorphism; A= adenine; C= cytosine; G= guanine; GpIIIa= glycoprotein IIIa; H= histidine; R= arginine; rs= reference sequence; T= thymine.

**Descriptive statistic and association analyses for the Beta-Fibrinogen G455A polymorphism.**

**Table S2a** - Frequency of G/A compared to G/G between cases and controls (Beta-Fibrinogen G455A

polymorphism).

| *SNP* | *Beta-Fibrinogen genotype* | *Case/Control (N freq)* | *P*  *value*  *(chi square)* |
| --- | --- | --- | --- |
| Beta-Fibrinogen G455A  rs#1800790 | GG | 63 (63.64%)/  38 (56.72 %) | 0.370 |
|  | GA | 36 (36.36%)/  29 (43.28 %) |  |

SNP: Single Nucleotide Polymorphism A= adenine; C= cytosine; G= guanine; rs= reference sequence.

**Table S2b** – Association analyses (multivariate logistic regression) between the Beta-Fibrinogen G455A polymorphism and MS status.

| *Model* | *Beta-Fibrinogen genotype* | *Adjusted OR* | *p value* | *[95% conf. Interval]* |
| --- | --- | --- | --- | --- |
| Additive model  (GG base outcome) | GA | 0.7475614 | 0.371 | .3953922- 1.413402 |
|  | AA | 8.050684 | **0.049** | 1.011232- 64.09361 |
| Recessive model  (GG+GA base outcome) | AA | 8.994 | **0.040** | 1,108 – 72,976 |

SNP: Single Nucleotide Polymorphism A= adenine; G= guanine; OR: Odds ratio; rs= reference sequence.

**Table S3.** Allele frequencies in European and Italian populations and in our cohorts of cases and controls.

| Hemostatic risk allele | rs number | Rs Allele Freq in European population | Rs Allele Freq in Italian population (TuscanY) | Rs Allele Freq in CamPania POpulation^#^ | Rs Allele Freq  in Ms patiens | Rs Allele Freq  in healthy controls |
| --- | --- | --- | --- | --- | --- | --- |
| B-fibrinogen G455A | rs1800790 | G: 78.43%,  A: 21.57% | G: 78.04%,  A: 21.96% | G: 80,1%,  A: 19.9% | G: 71.68%,  A: 28.3% | G: 77.21%  A: 22.79% |
| GpIIB/IIIa PIA2 | rs5918 | T: 86.78%,  C: 13.22% | T: 84.58%,  C: 15.42% | N/A | T: 80.1%,  C: 19.9% | T: 87.1%,  C: 12.9% |
| Factor V Leiden | rs6025 | G: 98.81%,  A: 1.19% | G: 99.53%,  A: 0.47% | G: 97.6%,  A: 2.4% | G: 97.9%,  A: 2.1% | G: 97.79%,  A:2.21% |
| Factor V H2R | rs1800595 | T: 93.84%,  C: 6.16% | T: 92.06%,  C: 7.94% | T: 95%,  C: 5% | T: 91.53%,  C: 8.47% | T: 96.97%,  C: 3.03% |
| Prothrombin G20210A | rs1799963 | G: 99.2%,  A: 0.8% | G: 97.2%,  A: 2.8% | G: 97.3%,  A: 2.7% | G: 95.8%,  A: 4.2% | G: 98.53%,  A: 1.47% |

A= adenine; C= cytosine; FREQ= frequency; G= guanine; GpIIIa= glycoprotein IIIa; MS= multiple sclerosis; RS= reference sequence; T= thymine. Source: Alexander, T.A., Machiela, M.J. LDpop: an interactive online tool to calculate and visualize geographic LD patterns. *BMC Bioinformatics* **21,**14 (2020). - <https://ldlink.nci.nih.gov/?var1=rs1799963&var2=rs1365120&pop=TSI&r2_d=r2&tab=ldpop>.

^#^ For population of Campania, source: Cernera G, Comegna M, Gelzo M, Savoia M, Bruzzese D, Mormile M, Zarrilli F, Amato F, Micco PD, Castaldo G. Molecular Analysis of Prothrombotic Gene Variants in Patients with Acute Ischemic Stroke and with Transient Ischemic Attack. *Medicina*. 2021; 57(7):723.

**Linkage disequilibrium**

We have verified whether the 5 tested SNPs [FGB 455 G/A (rs#1800790), Factor V 1691 G/A We have verified whether the 5 tested SNPs [FGB 455 G/A (rs#1800790), Factor V 1691 G/A (rs#6025), Factor V 1299 H/R; (#rs770011773), prothrombin 20210 G/A (rs#1799963); GpIIIa 1565 T/C (#rs59189)] were in linkage disequilibrium with some of the over 200 loci associated with MS.

We used published table in supplemental materials in the Science paper by the IMSGC (International Multiple Sclerosis Genetics Consortium. Multiple sclerosis genomic map implicates peripheral immune cells and microglia in susceptibility. Science. 2019 Sep 27;365(6460):eaav7188).

None of the 5 tested SNPs [FGB 455 G/A (rs#1800790) on chromosome 4; GpIIIa 1565 T/C (#rs59189) on chromosome 17; Factor V 1691 G/A (rs#6025) and Factor V 1299 H/R(#rs770011773) on chromosome 1; prothrombin 20210 G/A (rs#1799963) on chromosome 11] is in linkage disequilibrium with some of the 200 loci known to be associated with MS.

To investigate if the 5 tested SNPs are in LD with 200 loci described to be associated to MS, we used LDpop tool: <https://ldlink.nci.nih.gov/?tab=ldpop>. Details are reported in Table S4, S5, S6, S7 and S8.

**Table S4.** Investigate linkage disequilibrium patterns across B-fibrinogen (rs1800790) and loci on chromosome 4 described to be associated to Multiple sclerosis.

| rs number | Rs Allele Freq | R^2^ |
| --- | --- | --- |
| rs6533052 | G: 55.14%, A: 44.86% | 0 |
| rs2705616 | C: 50.47%, G: 49.53% | 0.0015 |
| rs6837324 | A: 65.42%, G: 34.58% | 0.0186 |
| rs9992763 | G: 35.98%, T: 64.02% | 0.0047 |
| rs2726479 | T: 45.79%, C: 54.21% | 0.0012 |
| rs17051321 | C: 76.64%, T: 23.36% | 0.0007 |
| rs13136820 | C: 38.79%, T: 61.21% | 0.0056 |
| rs72989863 | G: 59.35%, A: 40.65% | 0.0183 |

RS= reference sequence; R^2^= **R squared** measure of correlation of alleles for two genetic variants.

**Table S5.** Investigate linkage disequilibrium patterns across GpIIIa (rs5918) and loci on chromosome 17, described to be associated to Multiple Sclerosis.

| rs number | Rs Allele Freq | R^2^ |
| --- | --- | --- |
| rs2150879 | G: 44.86%, A: 55.14% | 0.001 |
| rs1026916 | A: 35.98%, G: 64.02% | 0.0033 |
| rs11079784 | T: 46.73%, C: 53.27% | 0.1038 |
| rs9909593 | A: 53.27%, G: 46.73% | 0.0045 |
| rs4796224 | A: 64.02%, G: 35.98% | 0.0026 |
| rs883871 | G: 89.25%, A: 10.75% | 0.0042 |
| rs7222450 | G: 60.75%, A: 39.25% | 0.0115 |
| rs9900529 | C: 33.64%, G: 66.36% | 0.0027 |

RS= reference sequence; R^2^= **R squared** measure of correlation of alleles for two genetic variants.

**Table S6.** Investigate linkage disequilibrium patterns across Factor V Leiden (rs6025) and loci on chromosome 1, described to be associated to Multiple Sclerosis.

| rs number | Rs Allele Freq | R^2^ |
| --- | --- | --- |
| rs10801908 | C: 86.92%, T: 13.08% | 0.0007 |
| rs6670198 | T: 69.16%, C: 30.84% | 0.0105 |
| rs1323292 | G: 19.16%, A: 80.84% | 0.0011 |
| rs35486093 | A: 92.52%, G: 7.48% | 0.0004 |
| rs11809700 | C: 73.36%, T: 26.64% | 0.0129 |
| rs34723276 | - | - |
| rs59655222 | T: 74.77%, C: 25.23% | 0.0016 |
| rs483180 | C: 70.09%, G: 29.91% | 0.002 |
| rs12133753 | C: 86.45%, T: 13.55% | 0.0007 |
| rs2317231 | G: 47.66%, T: 52.34% | 0.0052 |
| rs983494 | G: 79.44%, A: 20.56% | 0.0012 |
| rs72922276 | G: 95.79%, A: 4.21% | 0.0002 |
| chr1:32738415 | G: 90.65%, A: 9.35% | 0.0005 |
| rs1801133 | G: 53.27%, A: 46.73% | 0.0054 |
| rs58394161 | T: 79.91%, C: 20.09% | 0.0012 |
| rs3737798 | A: 48.13%, G: 51.87% | 0.0044 |
| rs9308424 | G: 60.28%, A: 39.72% | 0.0071 |
| rs6427540 | C: 89.25%, T: 10.75% | 0.0006 |
| rs6672420 | A: 42.99%, T: 57.01% | 0.0062 |
| rs11161550 | G: 50.47%, A: 49.53% | 0.0048 |
| chr1:154983036 | T: 95.33%, G: 4.67% | 0.0002 |
| rs11578655 | T: 83.64%, G: 16.36% | 0.024 |
| rs1415069 | G: 16.82%, C: 83.18% | 0.0009 |
| rs67934705 | - | - |
| rs198398 | T: 14.49%, C: 85.51% | 0.0008 |

RS= reference sequence; R^2^= **R squared** measure of correlation of alleles for two genetic variants.

**Table S7.** Investigate linkage disequilibrium patterns across Factor V H2R (rs1800595) and loci on chromosome 1, described to be associated to Multiple Sclerosis.

| rs number | Rs Allele Freq | R^2^ |
| --- | --- | --- |
| rs10801908 | C: 86.92%, T: 13.08% | 0.0039 |
| rs6670198 | T: 69.16%, C: 30.84% | 0.0022 |
| rs1323292 | G: 19.16%, A: 80.84% | 0.0011 |
| rs35486093 | A: 92.52%, G: 7.48% | 0.0023 |
| rs11809700 | C: 73.36%, T: 26.64% | 0.0004 |
| rs34723276 | - |  |
| rs59655222 | T: 74.77%, C: 25.23% | 0.0026 |
| rs483180 | C: 70.09%, G: 29.91% | 0.0344 |
| rs12133753 | C: 86.45%, T: 13.55% | 0.0002 |
| rs2317231 | G: 47.66%, T: 52.34% | 0.0043 |
| rs983494 | G: 79.44%, A: 20.56% | 0.0004 |
| rs72922276 | G: 95.79%, A: 4.21% | 0.0122 |
| chr1:32738415 | G: 90.65%, A: 9.35% | 0.0006 |
| rs1801133 | G: 53.27%, A: 46.73% | 0.0011 |
| rs58394161 | T: 79.91%, C: 20.09% | 0.0047 |
| rs3737798 | A: 48.13%, G: 51.87% | 0.0121 |
| rs9308424 | G: 60.28%, A: 39.72% | 0.0038 |
| rs6427540 | C: 89.25%, T: 10.75% | 0.0043 |
| rs6672420 | A: 42.99%, T: 57.01% | 0.0021 |
| rs11161550 | G: 50.47%, A: 49.53% | 0.0004 |
| chr1:154983036 | T: 95.33%, G: 4.67% | 0.0097 |
| rs11578655 | T: 83.64%, G: 16.36% | 0.0108 |
| rs1415069 | G: 16.82%, C: 83.18% | 0.021 |
| rs67934705 | - | - |
| rs198398 | T: 14.49%, C: 85.51% | 0.0005 |

RS= reference sequence; R^2^= **R squared** measure of correlation of alleles for two genetic variants.

**Table S8. I**nvestigate linkage disequilibrium patterns across Prothrombin (rs1799963) and loci on chromosome 11, described to be associated to Multiple Sclerosis.

| rs number | Rs Allele Freq | R^2^ |
| --- | --- | --- |
| rs4939490 | C: 55.61%, G: 44.39% | 0.0058 |
| rs6589706 | A: 42.06%, G: 57.94% | 0.0072 |
| rs12365699 | G: 88.32%, A: 11.68% | 0.0007 |
| rs34026809 | G: 94.39%, C: 5.61% | 0.0419 |
| rs2269434 | T: 73.83%, C: 26.17% | 0.0013 |
| rs6589939 | A: 61.68%, G: 38.32% | 0.0098 |
| rs4262739 | G: 59.81%, A: 40.19% | 0.0012 |
| rs4409785 | T: 80.84%, C: 19.16% | 0.0001 |
| chr11:14868316 | A: 98.13%, G: 1.87% | 0.0005 |
| rs11231749 | T: 69.16%, C: 30.84% | 0.0001 |
| chr11:118783424 | G: 97.2%, A: 2.8% | 0.0203 |
| rs56095240 | T: 81.31%, A: 18.69% | 0.0041 |
| rs531612 | C: 45.79%, T: 54.21% | 0.0002 |
| rs61884005 | C: 82.71%, G: 17.29% | 0.006 |
| rs35218683 | - | - |
| rs1365120 | C: 12.62%, T: 87.38% | 0.0004 |

RS= reference sequence; R^2^= **R squared** measure of correlation of alleles for two genetic variants.
